# FOXO3a Alleviates the Inflammation and Oxidative Stress *via* Regulating TGF-β and HO-1 in Ankylosing Spondylitis

**DOI:** 10.3389/fimmu.2022.935534

**Published:** 2022-06-17

**Authors:** Shanshan Xu, Xiaoyi Zhang, Yubo Ma, Yuting Chen, Huimin Xie, Lingxiang Yu, Jinian Wang, Sheng–qian Xu, Faming Pan

**Affiliations:** ^1^ Department of Epidemiology and Biostatistics, School of Public Health, Anhui Medical University, Hefei, China; ^2^ Inflammation and Immune Mediated Diseases Laboratory of Anhui Province, Hefei, China; ^3^ Department of Toxicology, School of Public Health, Anhui Medical University, Hefei, China; ^4^ Department of Hospital Management Research, The First Affiliated Hospital of Anhui Medical University, Hefei, China; ^5^ Department of Rheumatism and Immunity, The First Affiliated Hospital of Anhui Medical University, Hefei, China

**Keywords:** ankylosing spondylitis, FOXO3a, TGF-β, HO-1, inflammation, oxidative stress

## Abstract

This study aimed to investigate whether Forkhead box O3a (FOXO3a) modulates inflammation and oxidative stress in ankylosing spondylitis (AS). We applied bioinformatics analysis, quantitative real-time polymerase chain reaction, immunoblotting, enzyme linked immunosorbent assay, chromatin immunoprecipitation, and dual-luciferase reporter assay. Gene overexpression and knockdown of FOXO3a were conducted *via* lentivirus and small interfering RNA, respectively. Downregulated FOXO3a expression was first confirmed in AS patients. Interleukin-8 (IL-8) and IL-17A were highly expressed and negatively related with FOXO3a in AS. Total antioxidant capacity (T-AOC) were markedly decreased and positively associated with FOXO3a in AS. Overexpression of FOXO3a inhibited the secretion of inflammatory cytokines and promoted the production of antioxidant enzymes in Jurkat cells. Transforming growth factor-β (TGF-β) and heme oxygenase 1 (HO-1), which had binding sites to FOXO3a based on bioinformatics analysis, were abnormally expressed and positively related with FOXO3a. Accordingly, FOXO3a obviously elevated the protein and transcription levels of TGF-β and HO-1 in Jurkat cells. The above results were verified by silencing FOXO3a. Moreover, FOXO3a directly interacted with and promoted the transcription of TGF-β and HO-1. In summary, the modulation of cellular inflammation and oxidative stress *via* FOXO3a-mediated TGF-β and HO-1 activation is partly involved in the pathogenesis of AS.

**Graphical Abstract:**
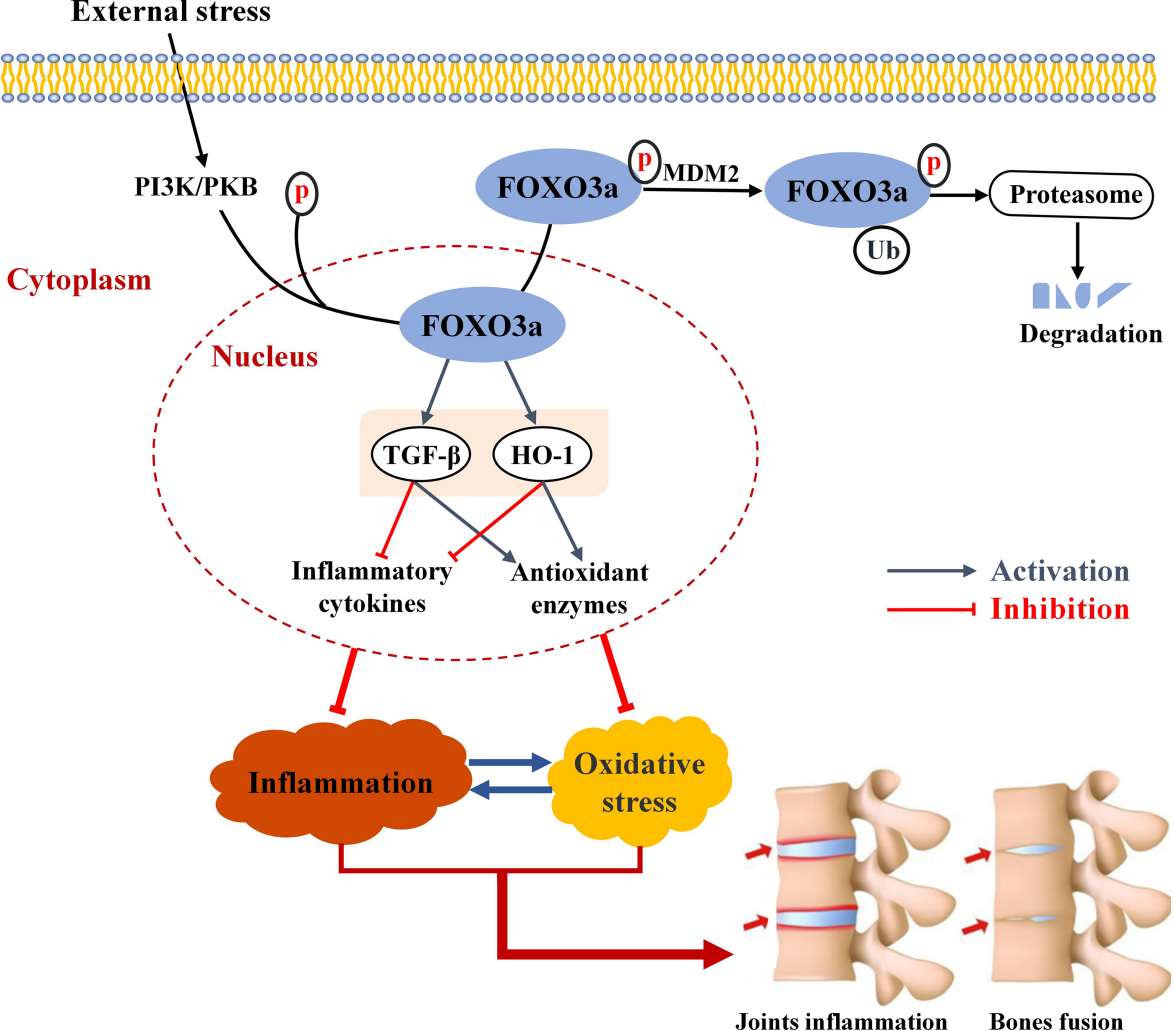
Schematic summary of FOXO3a in this study. In response to external stress, FOXO3a is phosphorylated, the most common post-translational modification, and discharged from the nucleus and degraded. FOXO3a directly interacts with TGF-β and HO-1 and activates TGF-β and HO-1, and reduces inflammatory cytokines and elevates antioxidant enzymes. The interaction between inflammation and oxidative stress can exacerbate joint inflammation and eventually bone erosion.

## Introduction

Ankylosing spondylitis (AS) is a chronic autoimmune inflammatory disease with a global prevalence of 0.2%-1.4%, mostly occurring in young men (1). AS patients are mainly manifested axial and peripheral joint pain and bone fusion, which lead to limited mobility and even disability, bringing heavy burden to patients, their families and society (2). AS is clinically incurable (3) and highly disabling (2), which has been considered as a major public health problem. Although the genetic basis of AS is known (4, 5), the molecular mechanisms responsible for the development of AS remain poorly understood.

Recently, the role of oxidative stress (OS) in the pathogenesis of AS has attracted attention. Several epidemiological reports showed that, compared with healthy controls (HCs), reactive oxygen species (ROS) and total oxidant status (TOS) in AS patients were significantly increased, while total antioxidant capacity (T-AOC) and total antioxidant status (TAS) were significantly decreased (6, 7). Notably, OS has been shown to be strongly linked with inflammation (8). The overproduction of ROS results in the initiation of the inflammatory process leading to the synthesis and secretion of proinflammatory cytokines (9, 10). Meanwhile, OS drives inflammation by motivating the nuclear factor-kappa B (NF-κB) and tumor necrosis factor alpha (TNF-a) pathway, which activate lymphocytes and macrophages, further augmenting ROS production and antioxidant loss, thereby exacerbating OS (11, 12). On the other hand, OS can also promote regulatory T (Treg) cell apoptosis and T helper 17 (Th17) cell differentiation, resulting in Treg/Th17 imbalance and inflammation (13). In general, the mutual promotion of oxidative stress and inflammation is closely related to the pathogenesis of AS, but the accurate molecular mechanism remains largely unknown.

Forkhead box O3a (FOXO3a) transcription factor, the foremost member of Forkhead box O family, is bound up with cell proliferation, apoptosis, autophagy, OS and aging (14). Among them, OS has been confirmed to be involved in the occurrence and development of AS (15). Increasing data demonstrated that FOXO3a deficiency may lead to the spontaneous activation of autoreactive Th cell, subsequent production of Th1 and Th2 cytokines, and eventual induction of autoimmune syndrome (16, 17). Several epigenetic studies indicated that rs12212067 polymorphism of FOXO3a was associated with the prognosis, inflammation and disease activity of rheumatoid arthritis (RA) (18), as well as the susceptibility to AS (19). Several animal experiments showed that the collagen-induced arthritis (CIA) mice lacking FOXO3a had severer arthritis (18) and FOXO3a overexpression could attenuate arthritis (20). A recent meta-analysis demonstrated that FOXO3a was abnormally expressed in autoimmune diseases (21). These findings strongly support the regulatory role of FOXO3a in the development of inflammatory diseases. Transforming growth factor-β (TGF-β) is a multifunctional cytokine (22), and its role in the pathogenesis of AS has been clarified, especially by regulating bone metabolism and inflammatory response (23). Heme oxygenase 1 (HO-1) is a protective cytokine with antioxidant, anti-apoptotic and anti-inflammatory effects, and has been shown to be a potential therapeutic target for autoimmune diseases (24). Besides, HO-1 was found to be significantly up-regulated in serum of AS patients and significantly correlated with ossification related indicators, which may be a potential biomarker of AS bone metabolism (25). Hitherto, the functional regulation between FOXO3a and HO-1 has not been proved yet, but some studies have shown that FOXO3a can improve the symptoms of autoimmune colitis mice by up-regulating TGF-β expression (26). Additionally, an online-based transcription factor database (http://jaspar.genereg.net/) found that FOXO3a has binding sites with TGF-β and HO-1. Interleukin-1β (IL-1β), IL-8, IL-17A, IL-23, and TNF-a play important roles in the inflammatory process of AS (27–29), and superoxide dismutase (SOD), catalase (CAT), T-AOC and malondialdehyde (MDA) are also valuable markers of oxidative stress in AS (15).

In this study, we investigated the role of FOXO3a and its downstream signaling molecules in the inflammatory process and oxidative stress of AS. The results of the current study might shed more light on the enigma of inflammation and OS in AS and facilitate the improvement of therapeutic strategies for AS patients.

## Materials and Methods

### Patient and Control Recruitment

The study was approved by the Ethical Committee of Anhui Medical University and all procedures have complied with the 1964 Declaration of Helsinki. All the subjects were given an informed consent and were well told of the study protocol. A total of 50 AS patients and 50 healthy individuals matched by age and gender were included using individual matching. Detailed inclusion and exclusion criteria are set out in **Appendix 1**. Ten milliliters peripheral blood samples and demographic and clinical characteristics (patients only) were collected.

### Bioinformatics

FOXO3a downstream target genes were predicted from the CHEA Transcription Factor Targets dataset, the JASPAR Predicted Transcription Factor Targets dataset, the TRANSFAC Predicted Transcription Factor Targets dataset, the TRANSFAC Curated Transcription Factor Targets dataset, which are available in the Harmonizome database (http://amp.pharm.mssm.edu/Harmonizome) (30).

### Cell Isolation

The peripheral blood mononuclear cells (PBMCs) from peripheral blood of subjects were extracted using Ficoll-Hypaque density gradient centrifugation method. The plasma was harvested for inflammatory factors and oxidative stress indicators. CD3^+^ T cells were isolated from PBMCs using CD3 microbeads (BD Bioscience, USA).

### Cell Culture

Jurkat cell, a human T-cell line, was a common cell model for immune-related diseases. Human Jurkat cells were incubated in RPMI 1640 medium containing 10% fetal bovine serum (Invitrogen-Gibco, USA), 100 U/mL penicillin, and 100 U/mL streptomycin at 37 °C with 5% CO^2^. Jurkat cells were activated with anti-CD3 (1 µg/ml) plus anti-CD28 (1 µg/ml) antibodies (eBioScience, San Diego, CA, USA) for 24 h.

### Cell Transfection

Lv-FOXO3a and Si-FOXO3a were purchased from the Hanbio Company (Shanghai, China). Lv-FOXO3a was applied to upregulate the expression of FOXO3a, and three siRNA sequences (Si-FOXO3a-P1, Si-FOXO3a-P2, Si-FOXO3a-P3) were designed to downregulate the expression of FOXO3a. Jurkat cells were transfected with Lv-FOXO3a and Si-FOXO3a using Lipofectamine 3000 (Invitrogen, USA). The sequences of Lv-FOXO3a and Si-FOXO3a are listed in **Table S1**.

### RNA Isolation and Quantitative Real-Time PCR

Total cellular RNA was extracted using TRIzol according to the instructions of the manufacturer (Invitrogen, USA). The quantification and concentration of RNA were measured using NanoDrop™ 2000 Spectrophotometer (Thermo Fisher Scientific, Wilmington, DE, USA). Then RNA was reverse-transcribed into complementary DNA (cDNA) using a PrimeScript™ RT reagent kit (Takara Bio Inc., Japan). qRT-PCR was performed in the ABI ViiA7 real-time PCR system (Applied Biosystems, Foster City, CA, USA) by SYBR Premix Ex Taq II (Takara Bio, Japan). Relative expression levels were calculated using the 2^-ΔΔCt^ formula. All tests were performed at three biological repeats. The primer sequences were shown in **Table S2**.

### Enzyme-Linked Immunosorbent Assay

The levels of IL-1β, IL-8, IL-17A, IL-23, and TNF-a were measured in the plasma and Jurkat cell supernatants by human ELISA kits (CUSABIO Life Sciences, MD, USA) according to the manufacturer’s instructions. Optical density was measured at 450 nm using the microplate reader (Thermo Fisher Scientific, Waltham, MA, USA) and the concentration was calculated according to the standard curve.

### Biochemical Assays

Jurkat cells were washed with phosphate buffer saline (PBS) and centrifuged. The collected cells were crushed by ultrasonic wave. The plasma and the supernatants were used for the measurement of SOD, CAT, and T-AOC activities and MDA content with a microplate reader according to the protocol of the detection kit. The activity of SOD was measured by the xanthine oxidase method. The activity of CAT was detected by the visible spectrophotometer method. The activity of T-AOC was detected by the colorimetric method. The MDA was quantified by the thiobarbituric acid method. Triplicates were maintained for this experiment.

### Immunoblotting

Jurkat cell lysates were prepared using M-PER™ Mammalian Protein Extraction Reagent (Thermo Fisher Scientific, USA) containing protease inhibitor cocktail. The concentrations of protein were determined using the BCA Protein Assay Kit (Thermo Fisher Scientific, USA). Antibodies used included FOXO3a (Thermo Fisher Scientific, 720128), TGF-β (Cell Signaling Technology, 3709S), HO-1 (Cell Signaling Technology, 43966S), and GADPH (Cell Signaling Technology, 2118S). Finally, chemical chromogenic reactions were performed using an enhanced chemo-luminescence detection kit (Thermo Fisher Scientific, USA).

### Chromatin Immunoprecipitation Assay

EZ-Magna Chromatin Immunoprecipitation Kits (Millipore) was applied for the ChIP assay according to the user’s manual. Briefly, 1×10^7^ human embryonic kidney 293 T (HEK-293T) cells were cross-linked in 1% formaldehyde (Macklin, Shanghai, China). The cell lysates were broken by sonication to produce DNA fragments of 200~1000 bp using the Bioruptor (Diagenode). Then normal rabbit IgG (Cell Signaling Technology, 2729) and anti-FOXO3a (Thermo Fisher Scientific, 720128) were used for immunoprecipitation reaction. After reverse cross-linking and DNA purification, the enriched DNA was subjected to q-PCR analysis using SYBR Green (Thermo Fisher Scientific). qPCR products were identified by agarose gel electrophoresis (AGE). The primers amplified genomic fragments containing putative FOXO3a sites on the TGF-β or HO-1 promoter, which were predicted by JASPAR database (http://jaspar.genereg.net/). Meanwhile, the promoter activity of TGF-β and HO-1 was predicted in BDGP: Neural Network Promoter Prediction (https://www.fruitfly.org/seq_tools/promoter.html/) and Promoter 2.0 Prediction (http://www.cbs.dtu.dk/services/Promoter/). The primer sequences used in qPCR are shown in **Table S3**.

### Dual-Luciferase Reporter Assay

HEK-293T Cells were seeded in 24-well plates and were co-transfected with FOXO3a or negative control expression plasmids, Renilla luciferase reporter vector, and Firefly luciferase reporter vectors with wild-type or mutant TGF-β/HO-1 using Lipofectamin 3000 (Invitrogen, USA) according to the manufacturer’s protocol. After 48h, luciferase activities were measured using Dual-Luciferase Reporter Assay System (Promega).

### Statistical Analysis

Data analysis was performed in SPSS 23.0 (SPSS, Chicago, IL, USA) and statistical graphs were produced in GraphPad Prism 7. Quantitative data of normal distribution were expressed as mean ± standard error of mean (SEM) and analyzed by Student’s t-test or analysis of variance (ANOVA). Qualitative data intergroup analysis was performed by Chi-square test. Quantitative data of skewed distribution were expressed by median and inter-quartile range (IQR), and Mann-Whitney U test was used for comparison between two groups. Correlation analyses were conducted with spearman rank correlation and partial correlation analysis. All statistical tests were two sides and *P* < 0.05 was considered to be statistically significant.

## Results

### FOXO3a Is Down-Regulated and Correlated With TGF-β and HO-1 in AS Patients

**Figure 1:**
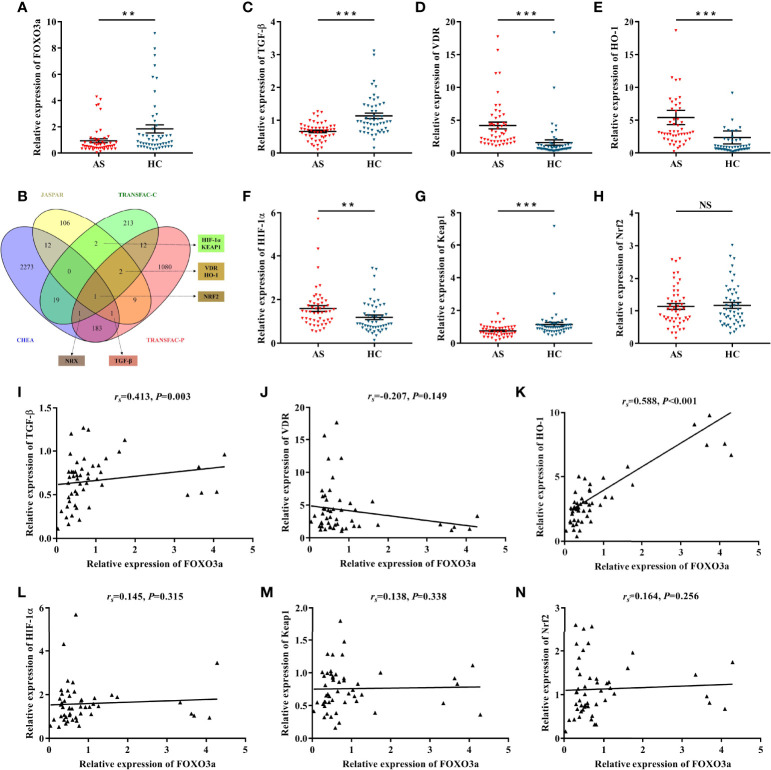
Determination of candidate genes and the correlation with FOXO3a. **(A)** Relative expression of FOXO3a between AS (n = 50) and HC (n = 50). **(B)** Predicting FOXO3a downstream target genes in different transcription factor datasets. Nucleoredoxin (NRX) was excluded because there is no evidence that it may be associated with AS and other autoimmune diseases. **(C–H)** Relative expression of TGF-β **(C)**, VDR **(D)**, HO-1 **(E)**, HIF-1α **(F)**, Keap1 **(G)**, and Nrf2 **(H)** between AS and HC. **(I–N)** Correlation analysis between FOXO3a and TGF-β **(I)**, VDR **(J)**, HO-1 **(K)**, HIF-1α **(L)**, Keap1 **(M)**, and Nrf2 **(N)** in AS. All data were expressed as *mean ± SEM*. The differences between AS and HC were analyzed using Mann-Whitney U test. The correlation was analyzed using spearman rank correlation. ^**^ *P* < 0.01, ^***^ *P* < 0.001; NS, not significant versus HC group. AS, ankylosing spondylitis; HC, healthy control; *r_s_*, Spearman’s rank correlation coefficient.

FOXO3a mRNA levels were determined in peripheral blood T cells of AS patient (n = 50) and healthy controls (n = 50). Clinical characteristics of subjects are listed in **Table 1**. As shown in **Figure 1A**, FOXO3a expression was significantly lower in AS patients than in controls. To analyze the potential regulatory mechanism of FOXO3a, we took the intersection of multiple datasets and combined with literature, genes that have binding sites to FOXO3a and may be related to the pathogenesis of AS were screened out, namely TGF-β, HO-1, vitamin D receptor (VDR), hypoxia-inducible factor-1α (HIF-1α), kelch-like ECH-associated protein 1 (KEAP1) and nuclear factor erythroid 2-related factor 2 (NRF2) (**Figure 1B**). Nucleoredoxin (NRX) was excluded because there is no evidence that it may be associated with AS and other autoimmune diseases. Among the predicted downstream target genes, except NRF2, all genes differentially expressed between AS patients and controls (**Figures 1C–H**). However, FOXO3a expression was only positively correlated with TGF-β and HO-1 expression in AS patients (**Figures 1I–N**). In addition, we further analyzed the correlation between FOXO3a, TGF-β, HO-1 and the main clinical features of AS patients. As presented in **Table 2**, FOXO3a expression was negatively correlated with Bath Ankylosing Spondylitis Functional Index (BASFI) in AS patients. TGF-β expression was inversely associated with Bath Ankylosing Spondylitis Disease Activity Index (BASDAI). HO-1 expression was positively related to erythrocyte sedimentation rate (ESR), C-reactive protein (CRP) and Ankylosing Spondylitis Disease Activity Score (ASDAS). Thus, FOXO3a may be involved in the development of AS by regulating the expression of TGF-β and HO-1, which needs further validation.

**Table 1:** Demographic and clinical characteristics of subjects.

	AS (n = 50)	HC (n = 50)	*Z/*χ^2^	*P* value
**Demographic characteristics**				
Age (year)	34.00 (26.00-46.25)	34.00 (26.00-47.00)	-0.069	0.945
Gender (male, %)	37 (74.0%)	37 (74.0%)	<0.001	1.000
**Clinical characteristics**				
WBC (×10^9^/L)	6.42 (5.50-7.68)	–		
NEU (×10^9^/L)	3.90 (2.92-4.58)	–		
MON (×10^9^/L)	0.45 (0.35-0.61)	–		
PLT (×10^9^/L)	225.50 (207.75-267.75)	–		
LYM (×10^9^/L)	1.86 (1.61-2.27)	–		
RDW (fL)	42.05 (39.98-45.45)	–		
PCT (%)	6.42 (5.50-7.68)	–		
HLA-B27 (+, %)	49 (98.0%)	–		
ESR (mm/h)	12.00 (5.00-24.25)	–		
CRP (mg/L)	5.45 (2.33-14.25)	–		
BASDAI (cm)	1.60 (0.37-3.00)	–		
BASFI (cm)	0.30 (0.00-2.35)	–		
ASDAS (cm)	2.06 (1.73-2.62)	–		
Disease duration (month)^‡^	12.00 (6.00-51.00)	–		
Global pain (cm)	2.00 (0.00-5.00)			
Night pain (cm)	2.50 (0.00-5.00)			
FFD (cm)	8.00 (0.75-21.75)			
Chest expansion (cm)	3.00 (3.00-4.25)			
Schober test (cm)	7.50 (5.00-10.25)			
PWD (cm)	1.00 (0.00-2.00)			
Treatment (n, %)				
DMARDs	23 (46.0%)			
NSAIDs	27 (54.0%)			
Biologic agents	22 (44.0%)			

AS, ankylosing spondylitis; ASDAS, Ankylosing Spondylitis Disease Activity Score; BASDAI, Bath Ankylosing Spondylitis Disease Activity Index; BASFI, Bath Ankylosing Spondylitis Functional Index; CRP, C-reactive protein; DMARDs, disease-modifying antirheumatic drugs; ESR, erythrocyte sedimentation rate; FFD, Finger-floor distance; HC, healthy control; LYM, lymphocyte; MON, monocyte; NEU, neutrophil; NSAIDs, nonsteroidal anti-inflammatory drugs; PCT, plateletcrit; PLT, platelet; PWD, pillow wall distance; RDW, red cell distribution width; WBC, white blood cell; -, not available.

Statistical methods: Mann-Whitney U test and Chi-square test.

All data were expressed as median (inter-quartile range).

**Table 2:** Correlation between FOXO3a, TGF-β, HO-1 and clinical indicators of AS patients.

Indicators	FOXO3a		TGF-β		HO-1	
	*r_s_*	*P* value	*r_s_*	*P* value	*r_s_*	*P* value
ESR	0.115	0.425	-0.085	0.557	0.293	**0.039**
CRP	-0.057	0.696	-0.248	0.083	0.283	**0.046**
BASDAI	0.123	0.396	-0.290	**0.041**	0.244	0.088
BASFI	-0.704	**0.016**	0.068	0.641	0.151	0.297
ASDAS	-0.032	0.827	-0.068	0.641	0.366	**0.009**
Disease duration	-0.193	0.570	-0.198	0.167	-0.111	0.441

AS, ankylosing spondylitis; ASDAS, Ankylosing Spondylitis Disease Activity Score; BASDAI, Bath Ankylosing Spondylitis Disease Activity Index; BASFI, Bath Ankylosing Spondylitis Functional Index; CRP, C-reactive protein; ESR, erythrocyte sedimentation rate.

r_s_, Spearman's rank correlation coefficient.

Statistical methods: Spearman rank correlation.

P values with bold were considered statistically significant differences.

### FOXO3a Is Related to Inflammation and Oxidative Stress in AS Patients

**Figure 2:**
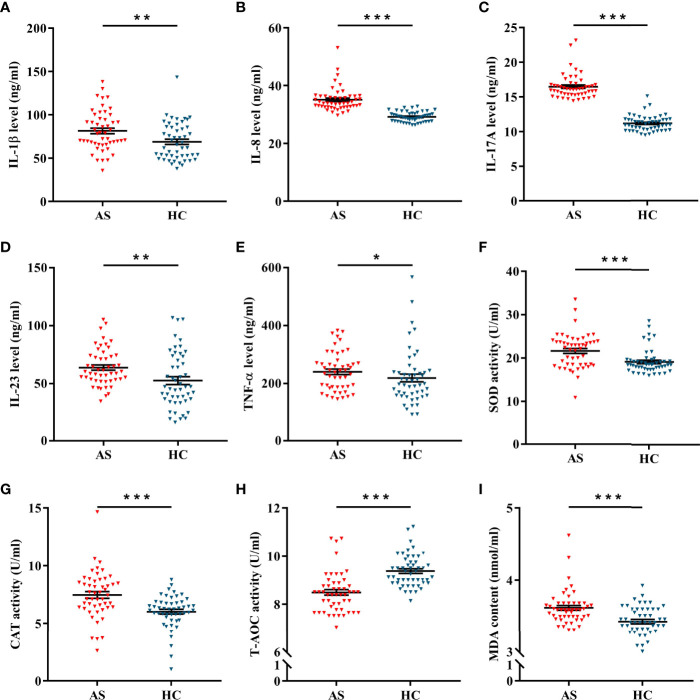
The levels of inflammation and oxidative stress in AS and HC. **(A–F)** The expression of IL-1β **(A)**, IL-8 **(B)**, IL-17A **(C)**, IL-23 **(D)** and TNF-α **(E)** between AS (n = 50) and HC (n = 50). **(F–H)** The activities of SOD **(F)**, CAT **(G)**, and T-AOC **(H)** between AS and HC. **(I)** The content of MDA between AS and HC. All data were expressed as *mean ± SEM*. The differences between AS and HC were analyzed using Mann-Whitney U test. ^*^ *P* < 0.05, ^**^ *P* < 0.01, ^***^ *P* < 0.001. AS, ankylosing spondylitis; HC, healthy control.

**Figure 3:**
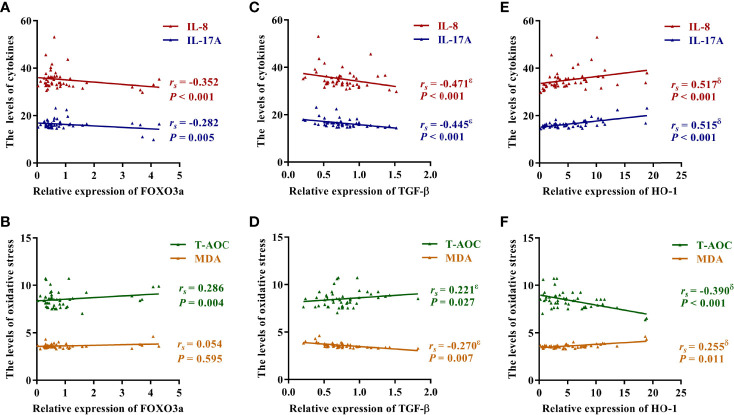
Correlation between FOXO3a, TGF-β, HO-1 and inflammation and oxidative stress. **(A)** Correlation between FOXO3a and IL-8 and IL-17A. **(B)** Correlation between FOXO3a and T-AOC and MDA. **(C)** Correlation between TGF-β and IL-8 and IL-17A. **(D)** Correlation between TGF-β and T-AOC and MDA. **(E)** Correlation between HO-1 and IL-8 and IL-17A. **(F)** Correlation between HO-1 and T-AOC and MDA. The correlation was analyzed using spearman rank correlation and partial correlation. *r_s_*, Spearman’s rank correlation coefficient; ϵ, the correlation was still significant in partial correlation analysis which controlling for HO-1; δ, the correlation was still significant in partial correlation analysis which controlling for TGF-β.

Inflammatory cytokines, antioxidant enzymes and lipid peroxides were detected in plasma samples of patients and controls. As shown in **Figures 2A–E**, the expression levels of IL-1β, IL-8, IL-17A, IL-23 and TNF-α were significantly elevated in AS patients. Of interest, the activities of SOD and CAT were obviously up-regulated in AS patients (**Figures 2F, G**), but T-AOC activity was obviously down-regulated (**Figure 2H**). Moreover, lipid peroxide levels, as measured by MDA content, were markedly enhanced in AS patients (**Figure 2I**). As presented in **Figures 3A, B**, FOXO3a level was negatively correlated with IL-8 and IL-17A levels, and positively correlated with T-AOC activity, but had no significant correlation with MDA content. Additionally, TGF-β and HO-1 were associated with IL-8, IL-17A, T-AOC, and MDA. (**Figures 3C–F**). Surprisingly, after controlling for either TGF-β or HO-1, the correlation between the other and indicators of inflammation and oxidative stress remained significant (**Table S4**). Moreover, **Table S5** showed that the levels of inflammation and T-AOC in AS patients were also related to the degree of disease activity. Taken together, these findings indicated that FOXO3a might regulate inflammatory responses and oxidative stress in AS patients, which provides a certain basis for the subsequent mechanism discussion.

### Overexpression and Knockdown of FOXO3a in Jurkat Cells

To explore the role of FOXO3a in the inflammation and oxidative stress, we altered the expression of FOXO3a in Jurkat cells. Firstly, lentivirus FOXO3a (Lv-FOXO3a), which was designed to improve the expression of FOXO3a, and negative control were respectively transfected into Jurkat cells. As shown in **Figure S1A**, Lv-FOXO3a significantly elevated the transcription level of FOXO3a. Secondly, Jurkat cells were transfected with three specific siRNA sequences (Si-FOXO3a-P1、Si-FOXO3a-P2、Si-FOXO3a-P3) to inhibit FOXO3a expression. As presented in **Figure S1B**, compared to the control group, Si-FOXO3a-P1 but not Si-FOXO3a-P2 or Si-FOXO3a-P3 obviously inhibited FOXO3a expression. Further, western blotting experiments were applied to verify the effectiveness of Lv-FOXO3a and Si-FOXO3a-P1. As shown in **Figures S1C, D**, the protein level of FOXO3a was markedly increased and decreased in Lv-FOXO3a group and Si-FOXO3a-P1 group, respectively. Combined with the above data, Lv-FOXO3a and Si-FOXO3a-P1 were selected to promote or inhibit FOXO3a expression in subsequent functional experiments.

### FOXO3a Regulates TGF-β and HO-1 in Jurkat Cells

**Figure 4:**
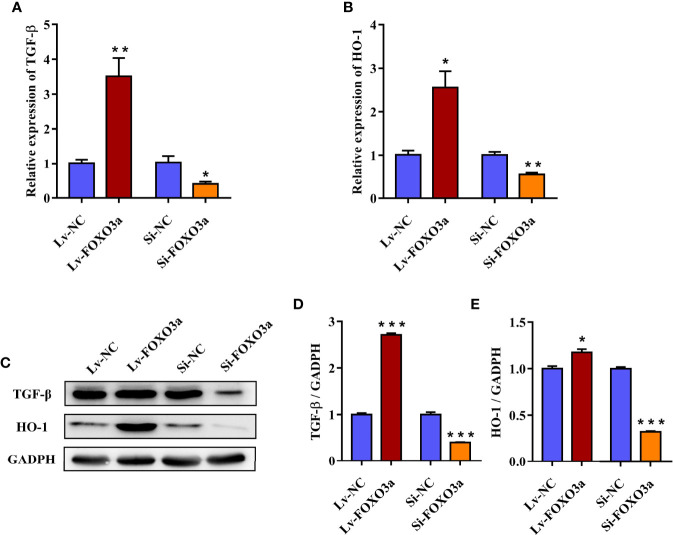
FOXO3a regulates TGF-β and HO-1 in Jurkat cells. **(A, B)** The mRNA levels of TGF-β and HO-1 were obviously increased by Lv-FOXO3a, but markedly decreased by Si-FOXO3a. **(C)** Representative immunoblots of TGF-β and HO-1 protein. **(D, E)** Quantification for TGF-β and HO-1. All data were expressed as *mean ± SEM* of three independent experiments (n = 3). The differences between experimental group and NC group were analyzed using Student’s t-test. ^*^ *P* < 0.05, ^**^ *P* < 0.01, ^***^ *P* < 0.001 versus NC group. Lv-NC, lentivirus negative control, Lv-FOXO3a, FOXO3a overexpression; Si-NC, siRNA negative control; Si-FOXO3a, FOXO3a knockdown.

To further investigate the effect of FOXO3a on TGF-β and HO-1, Jurkat cells were transfected with Lv-FOXO3a and Si-FOXO3a, and the transcription and protein levels of TGF-β and HO-1 were then measured. As shown in **Figures 4A, B**, the mRNA expression of TGF-β and HO-1 were significantly increased by Lv-FOXO3a and decreased by Si-FOXO3a. As expected, the above results also verified at the protein level. Lv-FOXO3a obviously elevated the protein expression of TGF-β and HO-1, and Si-FOXO3a inhibited their expression levels (**Figures 4C–E**). Altogether, these results confirmed that FOXO3a plays an important role in the development of AS by regulating TGF-β and HO-1.

### FOXO3a Modulates the Secretion of Inflammatory Cytokines in Jurkat Cells

**Figure 5:**
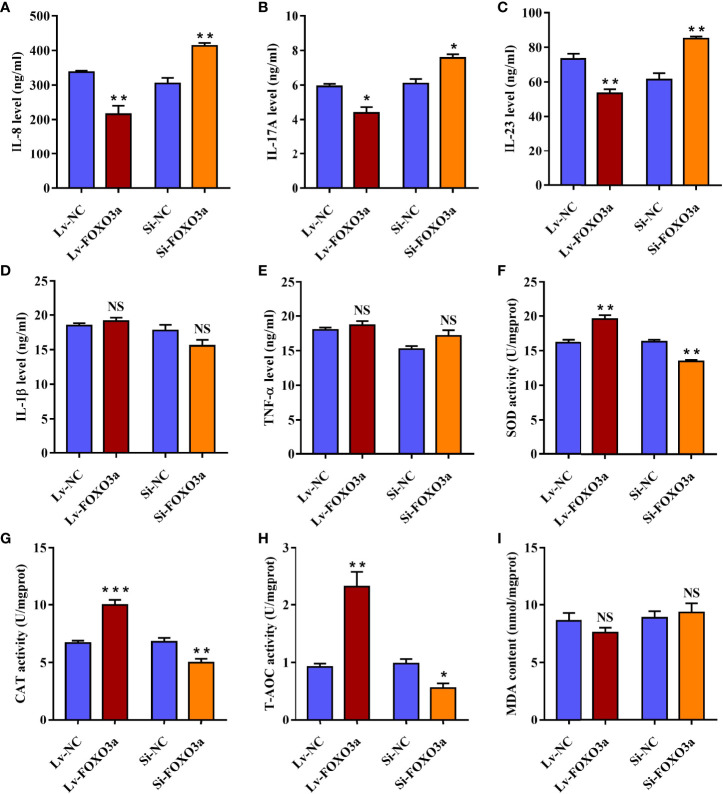
FOXO3a modulates inflammation and oxidative stress in Jurkat cells. **(A–E)** Lv-FOXO3a and Si-FOXO3a markedly altered the levels of IL-8 **(A)**, IL-17A **(B)**, and IL-23 **(C)**, and but had no significant effect on IL-1β **(D)** or TNF-α **(E)**. **(F–H)** Lv-FOXO3a obviously promoted the activities of SOD **(F)**, CAT **(G)**, and T-AOC **(H)**, which were obviously inhibited *via* Si-FOXO3a. **(I)** No remarkable difference on MDA was observed. All data were expressed as *mean ± SEM* of three independent experiments (n = 3). The differences between experimental group and NC group were analyzed using Student’s t-test. ^*^ *P* < 0.05, ^**^ *P* < 0.01, ^***^ *P* < 0.001; NS, not significant versus NC group. Lv-NC, lentivirus negative control, Lv-FOXO3a, FOXO3a overexpression; Si-NC, siRNA negative control; Si-FOXO3a, FOXO3a knockdown.

To verify the effect of FOXO3a on pivotal inflammatory cytokines in AS, the levels of IL-1β, IL-8, IL-17A, IL-23, and TNF-α cytokines in cell supernatants were then assessed. As shown in **Figures 5A–C**, Lv-FOXO3a obviously reduced the levels of IL-8, IL-17A, and IL-23 cytokines. Correspondingly, Si-FOXO3a markedly enhanced the levels of them. However, neither Lv-FOXO3a nor Si-FOXO3a significantly altered the levels of IL-1β and TNF-α cytokines (**Figures 5D, E**). Collectively, these findings support a role for FOXO3a in regulation of inflammatory responses, specifically affecting the release of IL-8, IL-17A, and IL-23 cytokine.

### FOXO3a Regulates the Levels of Antioxidant Enzymes in Jurkat Cells

In order to demonstrate the role of FOXO3a in oxidative stress, the activity of antioxidant enzymes and the content of MDA were analyzed in transfected Jurkat cells. As expected, the activities of SOD, CAT, and T-AOC were obviously elevated in Lv-FOXO3a group (**Figures 5F–H**). Nevertheless, the level of MDA was up-regulated slightly in Lv-FOXO3a group without statistically significant (**Figure 5I**). Similarly, Si-FOXO3a obviously decreased the activity of SOD, CAT, and T-AOC, but no remarkable difference on MDA was observed. Together, the above data suggested that FOXO3a plays a crucial role in alleviating oxidative stress, especially through the regulation of antioxidant enzymes.

### FOXO3a Directly Promotes TGF-β and HO-1 Genes Transcription

**Figure 6:**
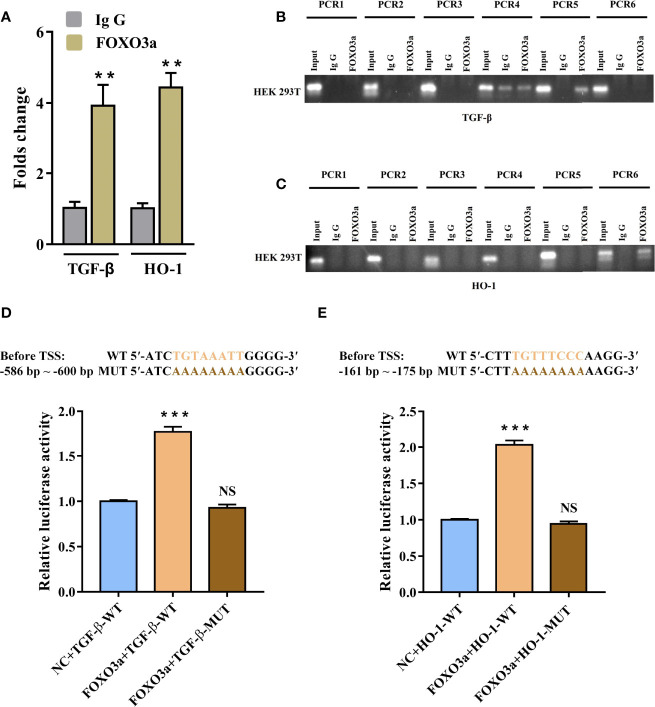
FOXO3a directly binds to TGF-β and HO-1 gene promoter. **(A–C)** Assessment of FOXO3a binding to TGF-β and HO-1 sequence in HEK-293 T cells was performed by ChIP-PCR. The fold change values were normalized to the negative control IgG. **(D–E)** Wild-type and mutant vectors of TGF-β **(D)** and HO-1 **(E)** based on the two positive binding sites in ChIP were synthetized. The luciferase activities of the constructs of TGF-β and HO-1 sequence in HEK-293 T cells transfected with either FOXO3a expression plasmid or controls. All data were expressed as *mean ± SEM* of three independent experiments (n = 3). The differences between experimental group and NC group were analyzed using Student’s t-test. ^**^ *P* < 0.01, ^***^ *P* < 0.001; NS, not significant versus NC group. TGF-β-WT, wild-type vectors of TGF-β; TGF-β-MUT, mutant vectors of TGF-β; HO-1-WT, wild-type vectors of HO-1; HO-1-MUT, mutant vectors of HO-1; NC, negative control.

To further confirm whether TGF-β and HO-1 are targets of FOXO3a, we used Chromatin immunoprecipitation assay (ChIP) and dual-luciferase reporter assays to verify it. **Figure S2** showed that the promoter regions of TGF-β and HO-1 had promoter activity. Meanwhile, JASPAR database predicted that both TGF-β and HO-1 gene promoters contained several potential binding sites of FOXO3a (**Table S6**). To identify the functional site of FOXO3a in TGF-β and HO-1 gene promoters, ChIP was used to pull down the FOXO3a-bound DNA in 293T cells. As shown in **Figure 6A**, compared with IgG immunoprecipitate, there were significant enrichment of two sequences (TGF-β: -590 to -597 bp before TSS; HO-1: -165 to -173 bp before TSS) in FOXO3a immunoprecipitate. The difference on the other sequences between two groups was not significant (**Table S7**). As expected, the agarose gel electrophoresis analysis verified that FOXO3a antibody effectively immunoprecipitated the above two sequences (**Figures 6B, C**). These results clarified that both the site from -590 to -597 bp of the TGF-β promoter and the site from -165 to -173 bp of the HO-1 promoter were essential for FOXO3a regulation.

To evaluate the ability and direction of FOXO3a on TGF-β and HO-1 gene transcription, we synthetized wild-type and mutant vectors of TGF-β and HO-1 based on the two positive binding sites in ChIP. As shown in **Figure 6D**, the 293 T cells co-transfected with FOXO3a expression plasmid and wild-type TGF-β vector had significantly higher luciferase activity compared with negative control. Similarly, as presented in **Figure 6E**, the transfection of the construct containing the FOXO3a expression plasmid and wild-type HO-1 vector led to a marked increase in the activity compared to that for the construct containing the wild-type HO-1 vector only. Additionally, we found that the mutants in the binding motif of TGF-β (“TGTAAATT”) and HO-1 (“TGTTTCCC”) could not affect the luciferase activity. These findings indicated that FOXO3a play a role in transcriptional regulation of TGF-β and HO-1. Overall, the above data further proved that FOXO3a transcriptionally activates TGF-β and HO-1 by directly binding to the promoter of TGF-β and HO-1.

## Discussion

AS is a kind of inflammatory autoimmune disease with complex pathogenesis. The existing genetic evidence still cannot fully explain the etiology of AS. Therefore, it is of vital importance to further explore the pathogenesis of AS from the aspects of molecular regulation and cellular function. A recent genetic study found that individuals carrying the rs12212067 G allele and rs3800232 T allele of FOXO3a had a significantly increased risk of AS, but did not clarify the role of FOXO3a in the pathogenesis of AS (19). This study aimed to investigate the specific mechanism by which FOXO3a regulates TGF-β and HO-1 to improve AS inflammation and oxidative stress. We firstly found that FOXO3a was significantly down-regulated in AS patients, and was significantly correlated with the indicators of inflammation and oxidative stress. Further *in vitro* experiments showed that FOXO3a could directly bind to TGF-β and HO-1 promoter regions and regulate the expression of corresponding genes, thereby inhibiting the release of inflammatory cytokines and promoting the expression of antioxidant enzymes.

FOXO3a has been identified as one of the most important tumor suppressors. With the deepening of the research on its abnormal expression in malignant tumors, FOXO3a has been found to play a pivotal role in the pathogenesis of autoimmune diseases (31). In the Human Protein Atlas (http://www.proteinatlas.org), FOXO3a expression in T cells is higher than that in other types of immune cells, such as B cells and PBMCs. And increasing studies reported that T cell subsets play an important role in the pathogenesis of AS (32, 33). Therefore, we measured FOXO3a expression in peripheral blood T cells of AS patients and found that it was obviously decreased. In addition, FOXO3a was negatively correlated with the inflammation level and BASFI of AS patients, suggesting that FOXO3a may be involved in the occurrence and development of AS. Meanwhile, the regulatory role of FOXO3a is bound to be closely related to cellular stress, cellular signal transduction, transcriptional initiation and regulation, and protein activation. Considering that FOXO3a is a multifunctional transcription factor, and its biological effect on regulating oxidative stress is related to the pathogenesis of AS (15). Then, we detected the levels of antioxidant enzymes and MDA, and found that oxidative stress existed in AS patients and was significantly correlated with FOXO3a expression. Among them, SOD and CAT were significantly up-regulated in AS patients, which was the result of the activation of human antioxidant defense system, while T-AOC level was significantly down-regulated, suggesting that there was strong oxidative stress in AS patients. A recent study showed that oxidative stress levels was associated with disease activity of AS (6), which is consistent with our findings. We found that T-AOC activity was negatively correlated with ESR, CRP and ASDAS. Therefore, FOXO3a may play a role in the occurrence and development of AS by regulating cellular inflammation and oxidative stress.

TGF-β, a cytokine that plays a dual role in the immune response, can not only inhibit the production and release of inflammatory cytokines, but also intensify the inflammatory response (34). Interestingly, TGF-β is mainly known for its regulation of peripheral tolerance *via* inhibiting the proliferation and differentiation of self-reactive T cells (35). It has been reported that TGF-β was markedly down-regulated in peripheral blood of AS patients (36), and significantly increased after treatment with etanercept (37), suggesting TGF-β plays a key role in the development of AS. HO-1, as an inducible antioxidant enzyme, is a classic marker of oxidative stress. HO-1 is stably low expressed in most tissues or cells under physiological conditions, but is up-regulated by NRF2 and activating protein-1 (AP-1) under pathological stress, indicating that HO-1 plays an important cellular protective role in inflammation and oxidative stress injury (38). Recent studies have shown that HO-1 was obviously overexpressed in RA, AS, and other autoimmune diseases (25), and could relieve inflammatory response, thus regulating related processes of bone metabolism (39). Based on bioinformatic analysis, we found FOXO3a can bind to TGF-β and HO-1 promoter transcription initiation regions. In addition, TGF-β was significantly decreased and HO-1 was markedly elevated in AS patients. Of note, the upregulation of HO-1 may well explain the enhancement of SOD and CAT in AS patients, which is the role of HO-1 as a cellular protective factor against oxidative stress, and can also be further evidence of active oxidative stress in AS patients. Furthermore, TGF-β and HO-1 were significantly correlated with FOXO3a expression, as well as inflammatory cytokines, oxidative stress status and clinical indicators in AS patients. Moreover, partial correlation results suggested that TGF-β and HO-1 were independently correlated with inflammation and oxidative stress. These results provide evidence that FOXO3a might modulate cellular inflammation and oxidative stress possibly through targeting TGF-β and HO-1.

In human monocytes, silencing FOXO3a significantly inhibited TGF-β production and subsequently regulated the production of proinflammatory and anti-inflammatory cytokine (40). It is well known that the expression of HO-1 is mainly directly regulated by NRF2 (38). The targeted regulation between FOXO3a and HO-1 has not been clarified yet. This study showed that the transcription and protein levels of TGF-β and HO-1 were elevated by Lv-FOXO3a and inhibited by Si-FOXO3a. Then ChIP and dual-luciferase reporter assays were used to verify the targeted regulation of FOXO3a on TGF-β and HO-1. The results indicated that FOXO3a promoted the transcription of TGF-β and HO-1 by binding to the -590~-597 bp before TSS of TGF-β and the -165~-173 bp before TSS of HO-1. This is the first time to elucidate the relationship between FOXO3a and TGF-β and HO-1 in Jurkat cells, expanding the role of FOXO3a in regulating downstream target genes and the pathway of TGF-β and HO-1 transcriptional regulation. All of above indicated that FOXO3a directly binds to the promoter area of TGF-β and HO-1 to activate the transcription.

Several studies demonstrated that TGF-β and HO-1 might be important regulators of the occurrence and development of AS and could modulate the inflammation and oxidative stress (25, 36). In this study, we measured the related indicators in Jurkat cells. The *in vitro* experiment showed that overexpression of FOXO3a significantly decreased the levels of IL-8, IL-17A and IL-23, and these results were confirmed by silencing FOXO3a. However, the effects of FOXO3a on IL-1β and TNF-α were not observed, suggesting that IL-8, IL-17A, and IL-23 play a key role in the inflammation progression of AS. Limón-Camacho and Mei et al. found that IL-8, IL-17A, and IL-23 were elevated in the serum of patients with AS (41, 42), which was consistent with the present study. Several studies have demonstrated that IL-23 can induce IL-17A production (43), and IL-17 collaborates with other inflammatory cytokines to increase IL-8 production (44), suggesting that IL-23/IL-17A axis and its regulation of IL-8 play a role in AS inflammation. Accumulating data suggest that FOXO3a activates antioxidant stress genes, such as SOD and CAT, to resist oxidative stress (45, 46). As expected, FOXO3a upregulated the activities of SOD, CAT and T-AOC, which were decreased after silencing FOXO3a. Of interest, HO-1, a marker of oxidative stress, was not increased but markedly decreased at low antioxidant levels caused by silencing FOXO3a, suggesting that FOXO3a has a greater regulatory effect on HO-1 than stress. Taken together, FOXO3a inhibits the release of IL-8, IL-17A, and IL-23, and promotes the production of antioxidant enzymes, thereby improving the inflammatory response and oxidative stress of AS.

In this study, our results suggest that FOXO3a directly binds and upregulates TGF-β and HO-1 to improve cellular inflammation and oxidative stress, and subsequently participates in the occurrence and development of AS. Recently, an epidemiological study found that a slightly increasement of mortality in AS patients (47), which is related to the socioeconomic status and complications. In this study, our results indicated that upregulation of FOXO3a improved cellular inflammation and oxidative stress. Moreover, the improvement in clinical symptoms in AS may be associated with up-regulation of FOXO3a (48). Meanwhile, the inactivation of FOXO3a is reversible, and the targeted therapy based on FOXO3a is theoretically easier to achieve. Therefore, reactivation of FOXO3a may provide a new therapeutic approach for AS, which has certain public health implications.

Our study pay attention to explore the effect of FOXO3a on inflammation and oxidative stress in AS patients. However, this study has several limitations. Firstly, the present study does not analyze the effect of FOXO3a on the bone metabolism of AS. Indeed, a recent study found that increased FOXO3a expression can prevent osteoblast differentiation and matrix calcification (49), and bone metabolism plays an important role in the pathogenesis of AS. Further study is necessary to explore the expression of FOXO3a in ligaments and bone marrow and the effects of FOXO3a on bone metabolism in AS patients. Secondly, this study lacked *in vivo* experiments. Our findings should be validated in AS animal models. Thirdly, this study did not simultaneously perform functional validation in primary T cells as well as in other immune cell lines, which needs to be implemented in future studies.

In summary, the present study analyzed the role of FOXO3a on inflammation and oxidative stress in AS. The case-control study showed that FOXO3a expression was obviously decreased and was associated with inflammation and total antioxidant capacity in AS patients. The *in vitro* experiment indicated that FOXO3a directly regulated TGF-β and HO-1 to alleviate inflammation and oxidative stress in Jurkat cells. These results provide evidence that the modulation of cellular inflammation and oxidative stress *via* FOXO3a mediated TGF-β and HO-1 activation partially takes part in the occurrence and development of AS (Graphical Abstract). Therefore, FOXO3a may serve as a potentially therapeutic target and provide clues to further understand the pathogenesis of AS.

## Data Availability Statement

The raw data supporting the conclusions of this article will be made available by the authors, without undue reservation.

## Ethics Statement

The studies involving human participants were reviewed and approved by Biomedical Ethics Committee of Anhui Medical University. The patients/participants provided their written informed consent to participate in this study.

## Author Contributions

SSX: Conceptualization; Data curation; Formal analysis; Investigation; Writing-original draft. XZ: Conceptualization; Formal analysis; Resources; Validation. YM: Methodology; Software; Visualization. YC: Data curation; Formal analysis. HX: Investigation. LY: Investigation. JW: Supervision; Resources. SQX: Resources; Writing-review & editing. FP: Conceptualization; Funding acquisition; Writing-review & editing. All authors contributed to the article and approved the submitted version

## Funding

This work was supported by the National Natural Science Foundation of China (82073655) and the Scientific Research Level upgrading Project of Anhui Medical University (2020xkjT006).

## Conflict of Interest

The authors declare that the research was conducted in the absence of any commercial or financial relationships that could be construed as a potential conflict of interest.

## Publisher’s Note

All claims expressed in this article are solely those of the authors and do not necessarily represent those of their affiliated organizations, or those of the publisher, the editors and the reviewers. Any product that may be evaluated in this article, or claim that may be made by its manufacturer, is not guaranteed or endorsed by the publisher.

## Supplementary Material

Supplementary Figure 1 Validation of FOXO3a overexpression and silencing. **(A)** Lv-FOXO3a significantly elevated the transcription level of FOXO3a. **(B)** Si-FOXO3a-P1 but not Si-FOXO3a-P2 or Si-FOXO3a-P3 obviously inhibited FOXO3a expression. **(C)** Representative immunoblots of FOXO3a protein. **(D)** Quantification for FOXO3a. All data were expressed as *mean ± SEM* of three independent experiments (n=3). The differences between experimental group and NC group were analyzed using Student’s t-test. ^*^ *P* < 0.05, ^**^ *P* < 0.01, ^***^ *P* < 0.001 versus NC group. Lv-NC, lentivirus negative control, Lv-FOXO3a, FOXO3a overexpression; Si-NC, siRNA negative control; Si-FOXO3a, FOXO3a knockdown.

Supplementary Figure 2 Analysis of candidate promoter region of TGF-β **(A, B)** and HO-1 **(C, D)** gene. **(A, B)** The promoter activity of TGF-β was predicted in BDGP: Neural Network Promoter Prediction **(A)** and Promoter 2.0 Prediction **(B)**. **(C, D)** The promoter activity of HO-1was predicted in BDGP: Neural Network Promoter Prediction **(C)** and Promoter 2.0 Prediction **(D)**. Both TGF-β and HO-1 promoter regions showed promoter activity7.

## Appendix

**Inclusion and exclusion criteria:** Patients meeting the following criteria were included: (1) the patients diagnosed by two rheumatologists according to the 1984 modified New York criteria; (2) the patients were not complicated with other autoimmune diseases, malignant tumors, neurodegenerative diseases, mental diseases, systemic infections or other chronic diseases; (3) the patients completed the epidemiological questionnaire survey of AS. Fifty AS patients were included from the outpatient department of the Department of Rheumatology and Immunology of the First Affiliated Hospital of Anhui Medical University from December 2020 to March 2021.

The selection criteria of healthy individuals were as follows: (1) no autoimmune diseases and chronic diseases related to infection; (2) no immediate family members had autoimmune diseases; (3) healthy in the past three months, without taking hormones or immunosuppressive drugs. Fifty healthy individuals were selected from the medical examination center of the First Affiliated Hospital of Anhui Medical University.
