# Supplementary figures and images for: FOXO3a Alleviates the Inflammation and Oxidative Stress via Regulating TGF-β and HO-1 in Ankylosing Spondylitis
Source: Front Immunol. 2022 Jun 17;13:935534. doi: 10.3389/fimmu.2022.935534 (PMC9247177; doi:10.3389/fimmu.2022.935534)

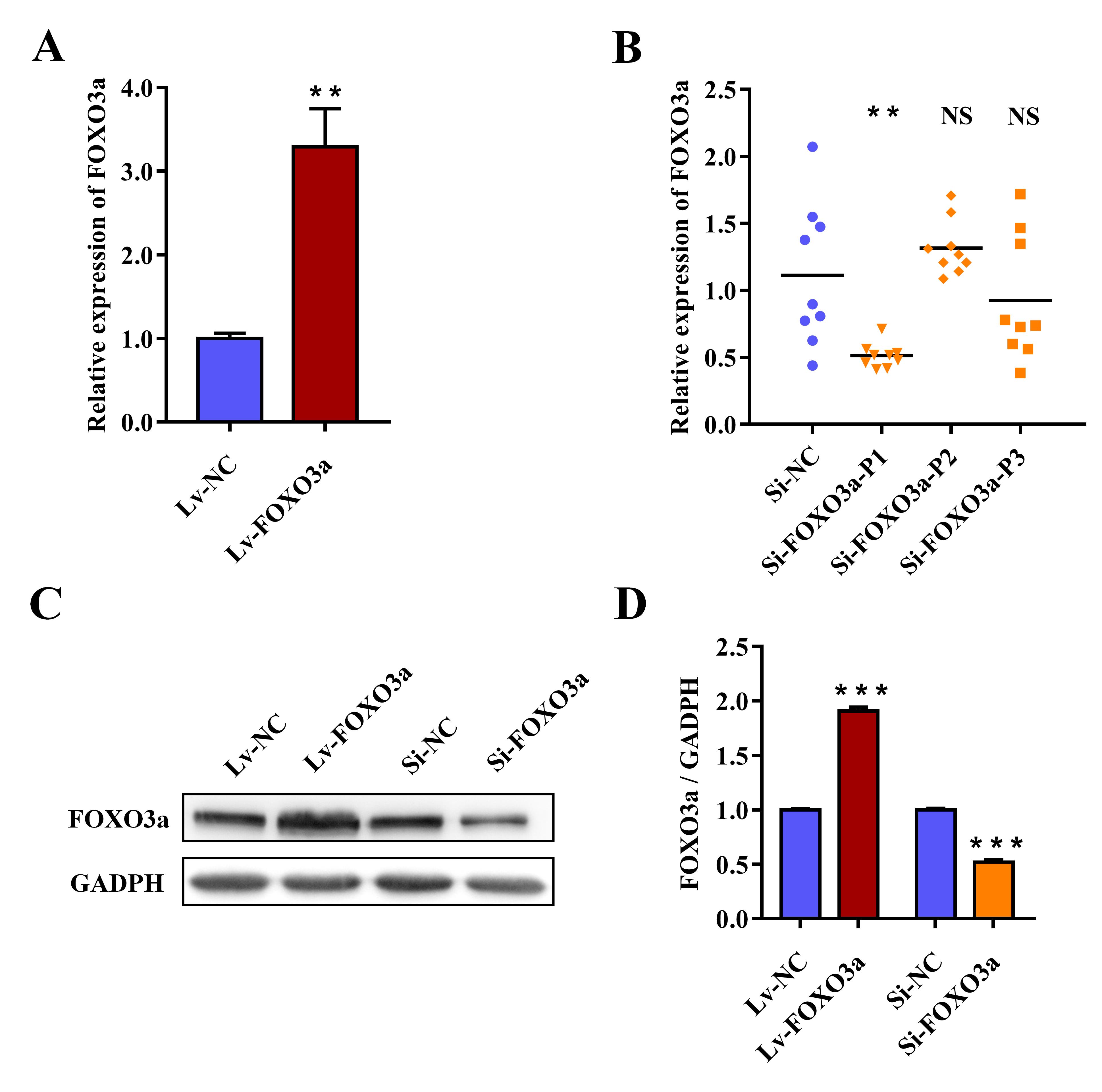

Supplement: Supplementary Figure 1 — Validation of FOXO3a overexpression and silencing. (A) Lv-FOXO3a significantly elevated the transcription level of FOXO3a. (B) Si-FOXO3a-P1 but not Si-FOXO3a-P2 or Si-FOXO3a-P3 obviously inhibited FOXO3a expression. (C) Representative immunoblots of FOXO3a protein. (D) Quantification for FOXO3a. All data were expressed as mean ± SEM of three independent experiments (n=3). The differences between experimental group and NC group were analyzed using Student’s t-test. * P < 0.05, ** P < 0.01, *** P < 0.001 versus NC group. Lv-NC, lentivirus negative control, Lv-FOXO3a, FOXO3a overexpression; Si-NC, siRNA negative control; Si-FOXO3a, FOXO3a knockdown. [file Image_1.jpeg]

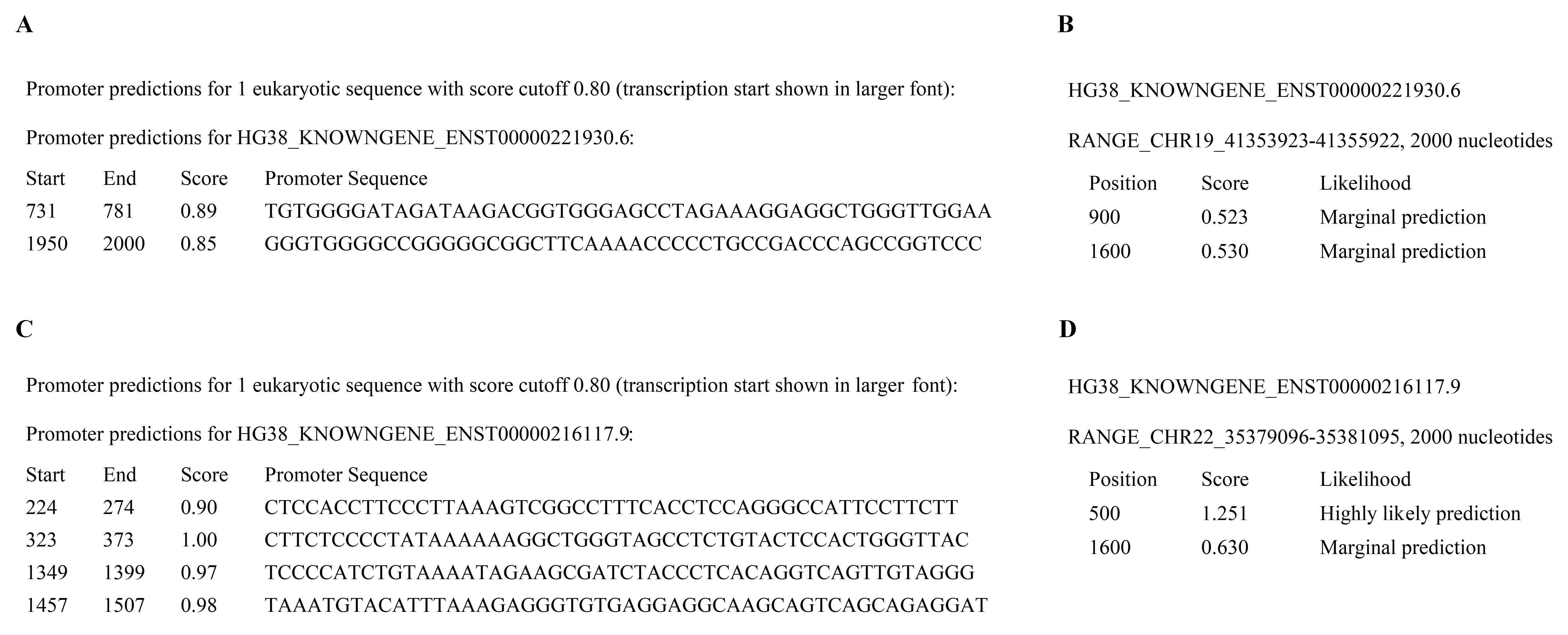

Supplement: Supplementary Figure 2 — Analysis of candidate promoter region of TGF-β (A, B) and HO-1 (C, D) gene. (A, B) The promoter activity of TGF-β was predicted in BDGP: Neural Network Promoter Prediction (A) and Promoter 2.0 Prediction (B). (C, D) The promoter activity of HO-1was predicted in BDGP: Neural Network Promoter Prediction (C) and Promoter 2.0 Prediction (D). Both TGF-β and HO-1 promoter regions showed promoter activity7. [file Image_2.jpeg]
