# Supplementary material for: FOXO3a Alleviates the Inflammation and Oxidative Stress via Regulating TGF-β and HO-1 in Ankylosing Spondylitis
Source: Front Immunol. 2022 Jun 17;13:935534. doi: 10.3389/fimmu.2022.935534 (PMC9247177; doi:10.3389/fimmu.2022.935534)
Supplement: Supplementary file 3 [file Table_1.docx]

Table S1 Sequences of Lv-FOXO3a and Si-FOXO3a

| Gene | Sense (5′-3′) | Antisense (5′-3′) |
| --- | --- | --- |
| Lv-FOXO3a | TAGAGGATCTATTTCCGGTGAATTCGCCACCATGGCAGAGGCACCGGCT | ACTTAAGCTTGGTACCGAGGATCCGCCTGGCACCCAGCTCTGAGATGAG |
| Lv-NC | Blank control | Blank control |
| Si-FOXO3a-P1 | ACUCCGGGUCCAGCUCCACTT | GUGGAGCUGGACCCGGAGUTT |
| Si-FOXO3a-P2 | CGUUCACGCACCAAUUCUATT | UAGAAUUGGUGCGUGAACGGA |
| Si-FOXO3a-P3 | GGAACGUGAUGCUUCGCAATT | UUGCGAAGCAUCACGUUCCGG |
| Si-NC | UUCUCCGAACGUGUCACGUTT | ACGUGACACGUUCGGAGAATT |
| Si-NC-FAM | UUCUCCGAACGUGUCACGUTT | ACGUGACACGUUCGGAGAATT-FA |

Lv, Lentivirus; NC, Negative control; Si, siRNA.
