# Supplementary material for: FOXO3a Alleviates the Inflammation and Oxidative Stress via Regulating TGF-β and HO-1 in Ankylosing Spondylitis
Source: Front Immunol. 2022 Jun 17;13:935534. doi: 10.3389/fimmu.2022.935534 (PMC9247177; doi:10.3389/fimmu.2022.935534)
Supplement: Supplementary file 4 [file Table_2.docx]

Table S2 Primer sequences for qRT-PCR

| Gene | Forward (5′-3′) | Reverse (5′-3′) |
| --- | --- | --- |
| FOXO3a | TCAAGGATAAGGGCGACAGC | GGACCCGCATGAATCGACTA |
| TGF-β | CTGTACATTGACTTCCGCAAG | TGTCCAGGCTCCAAATGTAG |
| VDR | AAAGGTCATTGGCTTTGCTAAG | CTTGACTTCAGCAGTACGATCT |
| HO-1 | CTTTCAGAAGGGCCAGGTGA | GTAGACAGGGGCGAAGACTG |
| HIF-1α | AATGCTCCCCTCACCCAACG | GCAGGGTCAGCACTACTTCG |
| NRF2 | CAGCTTTTGGCGCAGACATT | GACTGGGCTCTCGATGTGAC |
| KEAP1 | ATCGATGGCCACATCTATG | GATCCTTCGTGTCAGCATTG |
| β-actin | TCAGAGCAAGAGAGGCATCC | GTCATCTTCTCACGGTTGG |

β-actin was used as an internal control for mRNA.
