# Supplementary material for: FOXO3a Alleviates the Inflammation and Oxidative Stress via Regulating TGF-β and HO-1 in Ankylosing Spondylitis
Source: Front Immunol. 2022 Jun 17;13:935534. doi: 10.3389/fimmu.2022.935534 (PMC9247177; doi:10.3389/fimmu.2022.935534)
Supplement: Supplementary file 5 [file Table_3.docx]

Table S3 Primer sequence of ChIP-qPCR

| Gene | Promoter region (before TSS) | Primer sequence |
| --- | --- | --- |
| TGF-β | -1869 bp ~ -1876 bp | F1: 5′-TGAAGCTTCCTTGATCCCCT-3′ |
|  |  | R1: 5′-AGGGTAGATCAGATGGTGGT-3′ |
| TGF-β | -1820 bp ~ -1827 bp | F2: 5′-ACCACCATCTGATCTACCCT-3′ |
|  |  | R2: 5′-ACATGAACATGGATGGCAGACA-3′ |
| TGF-β | -1469 bp ~ -1477 bp | F3: 5′-GACATGAGGGAAGGCAGGAG-3′ |
|  |  | R3: 5′-ATCCTCCTCCATGACCCCAT-3′ |
| TGF-β | -1214 bp ~ -1224 bp | F4: 5′-AGATAAGACGGTGGGAGCCT-3′ |
|  |  | R4: 5′-TCCCCACTCCCTGATACTCA-3′ |
| TGF-β | -590 bp ~ -597 bp | F5: 5′-TCCTTTCTAGGACCTCGGGG-3′ |
|  |  | R5: 5′-CAGCCTCCTGTCACTCAACA-3′ |
| TGF-β | -383 bp ~ -417 bp | F6: 5′-GTGTCCTGTTGCCCCCTC-3′ |
|  |  | R6: 5′-ACCCAGAACGGAAGGAGAGT-3′ |
| HO-1 | -1660 bp ~ -1667 bp | F1: 5′-ACTGTCTTTCAAAAGAATTGTCTGCA-3′ |
|  |  | R1: 5′-GAATGGTGACCCTGTAACCCA-3′ |
| HO-1 | -1602 bp ~ -1606 bp | F2: 5′-GGTAGCCTCTGTACTCCACT-3′ |
|  |  | R2: 5′-ACCAACCGACAAAAGTCAGGT-3′ |
| HO-1 | -1503 bp ~ -1510 bp | F3: 5′-TGTATGTCTTTTCTCCTCTTAACCTG-3′ |
|  |  | R3: 5′-TCCCAAGGGTGAGCTAAGGG-3′ |
| HO-1 | -949 bp ~ -956 bp | F4: 5′-TCACAGTATTGGGAAAGGACTG-3′ |
|  |  | R4: 5′-TCTGCATGAGGTGGACTCCT-3′ |
| HO-1 | -565 bp ~ -593 bp | F5: 5′-TCTGCATGAGGTGGACTCCT-3′ |
|  |  | R5: 5′-TGGAATCCTCTGCTGACTGC-3′ |
| HO-1 | -165 bp ~ -173 bp | F6: 5′-TCCTTAAAGGTTTTGTGTGTGTGT-3′ |
|  |  | R6: 5′-CTTTCTGGCCGGGCGTTG-3′ |

F, Forward; R, Reverse; TSS, Transcription start site.
