# Supplementary material for: FOXO3a Alleviates the Inflammation and Oxidative Stress via Regulating TGF-β and HO-1 in Ankylosing Spondylitis
Source: Front Immunol. 2022 Jun 17;13:935534. doi: 10.3389/fimmu.2022.935534 (PMC9247177; doi:10.3389/fimmu.2022.935534)
Supplement: Supplementary file 6 [file Table_4.docx]

Table S4 Correlation between FOXO3a, TGF-β, HO-1 and inflammation and oxidative stress

| Indicators | FOXO3a | | | | | |  | TGF-β | | | |  | HO-1 | | | |
| --- | --- | --- | --- | --- | --- | --- | --- | --- | --- | --- | --- | --- | --- | --- | --- | --- |
|  | *r_s_* | *P* value | *r_p⸶_* | *P* value | *r_p⸶_* | *P* value |  | *r_s_* | *P* value | *r_p⸶_* | *P* value |  | *r_s_* | *P* value | *r_p⸷_* | *P* value |
| IL-1β | 0.050 | 0.621 | 0.068 | 0.503 | 0.084 | 0.409 |  | -0.075 | 0.456 | -0.147 | 0.144 |  | 0.045 | 0.653 | 0.056 | 0.577 |
| IL-8 | -0.352 | **<0.001** | -0.328 | **0.001** | -0.318 | **0.001** |  | -0.471 | **<0.001** | -0.441 | **<0.001** |  | 0.517 | **<0.001** | 0.488 | **<0.001** |
| IL-17A | -0.282 | **0.005** | -0.274 | **0.006** | -0.284 | **0.004** |  | -0.445 | **<0.001** | -0.434 | **<0.001** |  | 0.515 | **<0.001** | 0.496 | **<0.001** |
| IL-23 | 0.006 | 0.954 | 0.121 | 0.233 | 0.117 | 0.247 |  | -0.189 | 0.060 | -0.188 | 0.061 |  | 0.107 | 0.269 | 0.088 | 0.384 |
| TNF-α | -0.092 | 0.362 | 0.113 | 0.264 | 0.098 | 0.336 |  | -0.168 | 0.094 | -0.183 | 0.068 |  | 0.248 | **0.013** | 0.213 | **0.033** |
| SOD | -0.144 | 0.154 | -0.043 | 0.671 | -0.117 | 0.247 |  | -0.032 | 0.823 | -0.192 | 0.056 |  | 0.281 | **0.005** | 0.257 | **0.010** |
| CAT | -0.175 | 0.081 | -0.128 | 0.206 | -0.157 | 0.120 |  | -0.078 | 0.589 | -0.108 | 0.285 |  | 0.330 | **0.001** | 0.300 | **0.002** |
| T-AOC | 0.286 | **0.004** | 0.249 | **0.013** | 0.274 | **0.006** |  | 0.221 | **0.027** | 0.210 | **0.036** |  | -0.390 | **<0.001** | -0.349 | **<0.001** |
| MDA | 0.054 | 0.595 | 0.121 | 0.233 | 0.107 | 0.292 |  | -0.270 | **0.007** | -0.228 | **0.022** |  | 0.255 | **0.011** | 0.237 | **0.017** |

*r_s_*, Spearman's rank correlation coefficient; *r_p_*, partial correlation coefficient; *_⸶_*, the control variable is HO-1; *_⸷_*, the control variable is TGF-β.

Statistical methods: Spearman rank correlation and partial correlation analysis.

*P* values with bold were considered statistically significant differences.
