# Supplementary material for: FOXO3a Alleviates the Inflammation and Oxidative Stress via Regulating TGF-β and HO-1 in Ankylosing Spondylitis
Source: Front Immunol. 2022 Jun 17;13:935534. doi: 10.3389/fimmu.2022.935534 (PMC9247177; doi:10.3389/fimmu.2022.935534)
Supplement: Supplementary file 7 [file Table_5.docx]

Table S5 Correlation between indexes of inflammation and oxidative stress and clinical indicators

| Indicators | ESR | |  | CRP | |  | BASDAI | |  | BASFI | |  | ASDAS | |  | Disease duration | |
| --- | --- | --- | --- | --- | --- | --- | --- | --- | --- | --- | --- | --- | --- | --- | --- | --- | --- |
|  | *r_s_* | *P* value |  | *r_s_* | *P* value |  | *r_s_* | *P* value |  | *r_s_* | *P* value |  | *r_s_* | *P* value |  | *r_s_* | *P* value |
| IL-1β | -0.010 | 0.944 |  | -0.049 | 0.734 |  | 0.157 | 0.277 |  | 0.104 | 0.473 |  | 0.030 | 0.839 |  | 0.204 | 0.156 |
| IL-8 | 0.106 | 0.463 |  | 0.133 | 0.357 |  | 0.086 | 0.550 |  | 0.076 | 0.600 |  | 0.096 | 0.508 |  | 0.188 | 0.192 |
| IL-17A | 0.039 | 0.788 |  | 0.261 | 0.067 |  | 0.293 | **0.039** |  | 0.268 | 0.060 |  | -0.110 | 0.446 |  | 0.269 | 0.059 |
| IL-23 | 0.135 | 0.350 |  | 0.434 | **0.002** |  | 0.057 | 0.693 |  | 0.148 | 0.304 |  | 0.233 | 0.103 |  | 0.216 | 0.132 |
| TNF-α | 0.327 | **0.021** |  | 0.444 | **0.001** |  | 0.086 | 0.553 |  | 0.228 | 0.111 |  | 0.386 | **0.006** |  | -0.115 | 0.428 |
| SOD | -0.178 | 0.215 |  | -0.004 | 0.980 |  | 0.104 | 0.471 |  | 0.061 | 0.676 |  | 0.028 | 0.848 |  | 0.252 | 0.078 |
| CAT | -0.071 | 0.624 |  | -0.068 | 0.639 |  | 0.217 | 0.130 |  | 0.156 | 0.279 |  | 0.081 | 0.576 |  | 0.138 | 0.339 |
| T-AOC | -0.303 | **0.032** |  | -0.297 | **0.036** |  | -0.119 | 0.410 |  | 0.087 | 0.549 |  | -0.343 | **0.015** |  | -0.204 | 0.156 |
| MDA | -0.232 | 0.105 |  | -0.098 | 0.499 |  | 0.001 | 0.992 |  | 0.179 | 0.214 |  | -0.105 | 0.466 |  | 0.174 | 0.227 |

AS, ankylosing spondylitis; ASDAS, Ankylosing Spondylitis Disease Activity Score; BASDAI, Bath Ankylosing Spondylitis Disease Activity Index; BASFI, Bath Ankylosing Spondylitis Functional Index; CRP, C-reactive protein; ESR, erythrocyte sedimentation rate.

*r_s_*, Spearman's rank correlation coefficient.

Statistical methods: Spearman rank correlation.

*P* values with bold were considered statistically significant differences.
