# Supplementary material for: FOXO3a Alleviates the Inflammation and Oxidative Stress via Regulating TGF-β and HO-1 in Ankylosing Spondylitis
Source: Front Immunol. 2022 Jun 17;13:935534. doi: 10.3389/fimmu.2022.935534 (PMC9247177; doi:10.3389/fimmu.2022.935534)
Supplement: Supplementary file 8 [file Table_6.docx]

Table S6 FOXO3a binding sites with TGF-β and HO-1

| Matrix ID | Name | Score | Relative score | Gene | Start | End | Strand | Predicted sequence |
| --- | --- | --- | --- | --- | --- | --- | --- | --- |
| MA0157.1 | FOXO3a | 10.2054 | 0.934945143 | TGF-β | 124 | 131 | - | GGAAAACA |
| MA0157.2 | FOXO3a | 9.69352 | 0.902518451 | TGF-β | 1609 | 1616 | - | GGAAACAA |
| MA0157.1 | FOXO3a | 9.16847 | 0.900112458 | TGF-β | 1610 | 1617 | - | GGGAAACA |
| MA0157.2 | FOXO3a | 9.35868 | 0.895765621 | TGF-β | 1583 | 1590 | - | GTCAACAT |
| MA0157.2 | FOXO3a | 8.89585 | 0.886431602 | TGF-β | 523 | 530 | - | GTAACCAT |
| MA0157.2 | FOXO3a | 8.60383 | 0.88054217 | TGF-β | 173 | 180 | - | GTAAATAA |
| MA0157.1 | FOXO3a | 8.19509 | 0.867414607 | TGF-β | 1584 | 1591 | - | TGTCAACA |
| MA0157.1 | FOXO3a | 7.60212 | 0.847495372 | TGF-β | 524 | 531 | - | GGTAACCA |
| MA0157.1 | FOXO3a | 6.99905 | 0.827237182 | TGF-β | 776 | 783 | + | TGGAAACT |
| MA0157.1 | FOXO3a | 6.61723 | 0.814410977 | TGF-β | 1403 | 1410 | + | TGTAAATT |
| MA0157.2 | FOXO3a | 9.69352 | 0.902518450 | HO-1 | 1827 | 1834 | - | GGAAACAA |
| MA0157.1 | FOXO3a | 9.16847 | 0.9001124581 | HO-1 | 1828 | 1835 | - | GGGAAACA |
| MA0157.1 | FOXO3a | 8.03598 | 0.8620698506 | HO-1 | 1407 | 1414 | + | TGAAAATA |
| MA0157.1 | FOXO3a | 7.60212 | 0.8474953720 | HO-1 | 391 | 398 | - | GGTAATCA |
| MA0157.1 | FOXO3a | 7.49457 | 0.8438828712 | HO-1 | 1797 | 1804 | - | AGAAAACA |
| MA0157.1 | FOXO3a | 6.95915 | 0.8258969164 | HO-1 | 490 | 497 | - | TGAAAAAA |
| MA0157.2 | FOXO3a | 6.40607 | 0.8073179630 | HO-1 | 1044 | 1051 | + | GGGAAATA |
| MA0157.2 | FOXO3a | 5.51536 | 0.8182556306 | HO-1 | 333 | 340 | - | ATAAAAAA |
| MA0157.1 | FOXO3a | 5.21823 | 0.8122632910 | HO-1 | 1428 | 1435 | - | GTCAAAAA |
