# Supplementary material for: FOXO3a Alleviates the Inflammation and Oxidative Stress via Regulating TGF-β and HO-1 in Ankylosing Spondylitis
Source: Front Immunol. 2022 Jun 17;13:935534. doi: 10.3389/fimmu.2022.935534 (PMC9247177; doi:10.3389/fimmu.2022.935534)
Supplement: Supplementary file 9 [file Table_7.docx]

Table S7 Results of ChIP-qPCR detection

| Gene | PCR | Fold change | *t* | *P* value |
| --- | --- | --- | --- | --- |
| TGF-β | PCR1 | 1.698 ± 0.249 | -2.428 | 0.072 |
| TGF-β | PCR2 | 1.051 ± 0.140 | -0.151 | 0.887 |
| TGF-β | PCR3 | 1.226 ± 0.130 | -1.674 | 0.169 |
| TGF-β | PCR4 | 1.102 ± 0.221 | 0.554 | 0.633 |
| TGF-β | PCR5 | 3.921 ± 0.586 | -4.745 | **0.009** |
| TGF-β | PCR6 | 1.628 ± 0.304 | -2.001 | 0.172 |
| HO-1 | PCR1 | 0.976 ± 0.088 | 0.272 | 0.799 |
| HO-1 | PCR2 | 1.168 ± 0.186 | -0.548 | 0.613 |
| HO-1 | PCR3 | 1.208 ± 0.145 | -1.270 | 0.273 |
| HO-1 | PCR4 | 0.763 ± 0.152 | 0.811 | 0.463 |
| HO-1 | PCR5 | 0.983 ± 0.133 | 0.331 | 0.757 |
| HO-1 | PCR6 | 4.433 ± 0.411 | -7.882 | **0.001** |

Statistical methods: student's t-test.

All variables were presented as means ± standard error of mean (SEM);

*P* values with bold were considered statistically significant differences.
